# *In Vitro* Antimicrobial Effect of a Cold Plasma Jet against *Enterococcus faecalis* Biofilms

**DOI:** 10.5402/2012/295736

**Published:** 2012-01-24

**Authors:** Chunqi Jiang, Christoph Schaudinn, David E. Jaramillo, Paul Webster, J. William Costerton

**Affiliations:** ^1^Department of Electrical Engineering-Electrophysics, Viterbi School of Engineering, University of Southern California, Los Angeles, CA 90089, USA; ^2^Electron Microscopy and Advanced Imaging Center, House Ear Institute, Los Angeles, CA 90057, USA; ^3^Endodontic Department, School of Dentistry, Loma Linda University, Loma Linda, CA 92354, USA; ^4^Center for Genomic Sciences, Allegheny-Singer Research Institute, Pittsburgh, PA 15212, USA

## Abstract

The hypothesis that a cold plasma jet has the antimicrobial effect against *Enterococcus faecalis* biofilms was tested *in vitro*. 27 hydroxyapatite discs were incubated with *E. faecalis* for six days to form a monoculture biofilm on the disc surface. The prepared substrata were divided into three groups: the negative control, the positive control (5.25% NaOCl solution), and the plasma treatment group. Resultant colony-forming unit counts were associated with observations of bacterial cell morphology changes using scanning electron microscopy (SEM). Treatment of *E. faecalis* biofilm with the plasma and 5.25% NaOCl for 5 min resulted in 93.1% and 90.0% kill (*P* < 0.0001), respectively. SEM detected that nearly no intact bacteria were discernible for the plasma-exposed HA disc surfaces. The demonstrated bactericidal effect of the plasma with direct surface contact may be due to the enhanced oxidation by the locally produced reactive plasma species.

## 1. Introduction

The ultimate goal of endodontic treatment is to eliminate bacterial infection in the root canal system and allow healing of apical periodontitis [1, 2]. Most infecting bacteria and their principal substrate of necrotic pulp debris may be removed by routine intracanal procedures including mechanical instrumentation and the use of intra-canal irrigants and medicaments that have antimicrobial activities [3, 4]. However, persistent periradicular infection has been frequently observed after single-visit and multiple-visit root canal treatments [5, 6]. The survival of microorganisms in the apical portion of the root-filled tooth has been considered as the major cause of failure [7–9]. *Enterococcus faecalis*, a gram-positive facultative bacterium, is a frequent and persistent isolate in teeth with failed root canal therapy [10]. Although *E. faecalis* only makes up a small proportion of the flora in untreated canals, it plays a major role in the etiology of persistent periradicular lesions after root canal treatment [7, 8]. Its occurrence in root-filled teeth with periradicular lesions was reported in a prevalence range from 24 to 77% [2, 7, 8]. It has been suggested that microbial growth as biofilm may enable the microorganisms to survive harsh growth conditions such as encountered in the postendodontic root canal environment [11]. In a biofilm, bacteria are embedded in a self-produced extracellular polymeric matrix, forming a sessile microbial community. The altered bacterial phenotype, along with the biofilm matrix, makes the biofilm less susceptible to antibiotics and the host immune response [11, 12]. Bacterial biofilms are thereby considered to be a common cause of numerous oral infections including dental caries, pulpitis, periodontitis, and periradicular lesions [13, 14].

Recent development of nonthermal, atmospheric-pressure plasmas that can enter the root canal of teeth that have been drilled and cleaned has made it possible to use the plasma to remove the microorganisms associated with infected root canals [15]. Plasma, the fourth state of matter, is a quasineutral collection, consisting of neutral species and charged particles. A non-thermal, atmospheric-pressure plasma enhances the generation of reactive chemical species and the interaction of these species with the objects under treatment, while the bulk gas remains near room temperature. These properties have made these plasmas highly attractive in a variety of biomedical and environmental applications, including low-heat surface modification of polymers [16], clinical instrument sterilization [17], and food processing [18]. A room temperature plasma dental probe [15] was developed to generate a 3 cm long, 1-2 mm diameter plasma plume for root canal disinfection. The reactive plasma species (e.g., reactive oxygen species and charged species) can be introduced by the plasma plume and capable of penetrating everywhere in the root canal, including through dentinal tubules, and disinfect surfaces by bactericidal processes [15, 19]. In this *in vitro* study, the antimicrobial effect of a needle-shape, room-temperature plasma jet against *E. faecalis *biofilms was assessed.

## 2. Materials and Methods

### 2.1. *E. faecalis* Biofilm Formation

27 sintered hydroxyapatite (HA) discs (Clarkson Chromatography Products Inc. South Williamsport, PA, USA) were placed in lids, which were clipped from polyethylene BEEM Capsules (Size 00, Ted Pella, Redding, CA, USA). The HA discs, 9.5 mm in diameter and 2 mm thick, together with their lid-holders, were disinfected by immersing them in 6% NaOCl for 15 min and afterwards rinsed ten times in sterile double-distilled water (ddH_2_O). All cleaned HA discs were then bathed for 8 hours in filter-sterilized human-stimulated saliva to facilitate bacterial adhesion [20]. Triplicates of the HA discs were placed in six-well polystyrene cell culture plates (Greiner bio-one, Monroe, NC, USA), whereas each well was filled with 7.5 mL Luria Bertani (LB) broth (BD Diagnostic System, Sparks, MD, USA). 500 *μ*L of an overnight *E. faecalis *(ATCC 29212) culture (LB broth) was added to each well. The disc-containing six-well-plates were incubated aerobically at 37°C for 6 days. 5 mL of the culture media in each well was changed daily.

### 2.2. Plasma Treatment and the Controls

Prior to treatment, the discs in their holders were taken out from the six-well plates and the excess liquid was carefully removed with sterile filter paper. The 27 HA discs with *E. faecalis *biofilms were randomly divided into three groups (*n* = 9 in each group): the negative control group, the 5.25% NaOCl treatment group (positive control), and the plasma treatment group.

**Figure 1:**
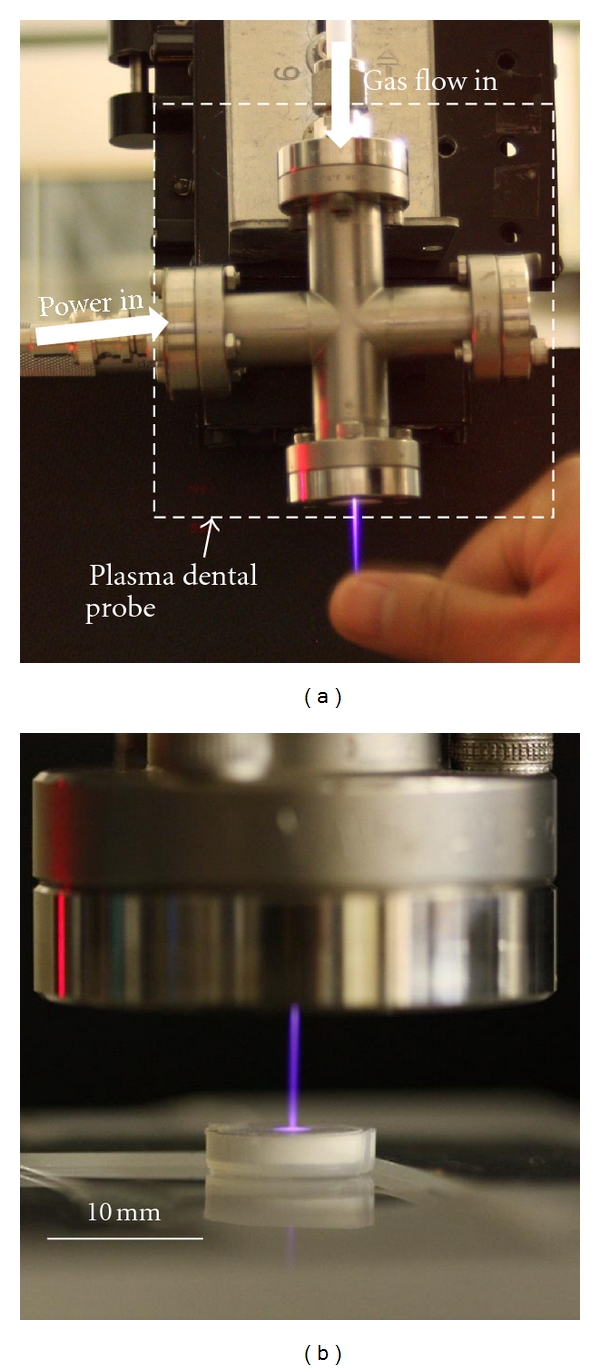
(a) A prototype of the plasma source generating a 1 mm diameter plasma plume that can be hand touched without causing pain or burning. (b) The plasma plume impinging to an HA disc, 1 cm below the plasma device nozzle.

The plasma was generated by a proprietary plasma device, a plasma dental probe [15], powered by 6 kV, ~100 ns voltage pulses at a rate of 1 kHz. The applied plasma plume was 1 mm in diameter and operated at an average power of 0.7 W. A laminar gas flow of premixed He/(1%)O_2_ mixture (Prepared by Airgas) at a flow rate of 1 SLPM (Standard Liter per Minute) sustains the plasma and provides the gas molecules for generation of reactive chemical species at room temperature. The plasma plume can be touched with hands without causing pain or burning, as shown in Figure 1(a). Since the plasma must be operated with a gas flow, the negative control group was treated with the same gas flow but with plasma switched off. The discs of both the negative control group and the plasma treatment group were placed 1 cm below the device nozzle for 5 min.  Figure 1(b) shows the plasma plume impinging to an HA disc. The discs of the positive control group were immersed in 8 mL 5.25% NaOCl for 5 min. Each disc of all the groups was gently rinsed in sterile LB broth (10 × 50 mL) immediately following the treatment.

### 2.3. Microbiological Statistical Analysis

After treatment, 15 randomly selected specimens (5 specimens per group) were used for the microbiological analysis. The number of colony-forming units (CFUs) was determined using the conventional culture techniques and dilution series. The discs were carefully removed from their holders and placed individually in Eppendorf tubes, containing 1 mL LB broth. Bacterial cells and biofilms were sheared off from HA discs by vigorously vortexing for 30 s, followed by ultrasonic treatment for another 30 s. All the samples were diluted appropriately, plated on LB agar plates, and incubated for 16 h at 37°C before counting. Average CFU values per volume and standard deviations from the experiments were obtained. The parametric test of one-way Analysis of Variance (ANOVA) was used to analyze statistical significance, and a *P* value of <0.05 was considered statistically significant.

### 2.4. Preparation for Scanning Electron Microscopy

The remaining 12 HA discs (*n* = 4 for each group) were immediately fixed in a solution of 4% formaldehyde and 2% glutaraldehyde in phosphate buffered saline (PBS) (pH 7.2) for 24 h at 4°C. The discs were then carefully washed in PBS, dehydrated in a graded ethanol series and critical point dried. Finally, they were mounted on a stub, sputter coated with a 25 nm layer of platinum and documented with SEM operating at 5 kV in the secondary electron mode (XL 30 S FEG, FEI Company, Hillsboro, OR).

## 3. Results

### 3.1. Viability of Residual Biofilm-Forming Cells

**Figure 2:**
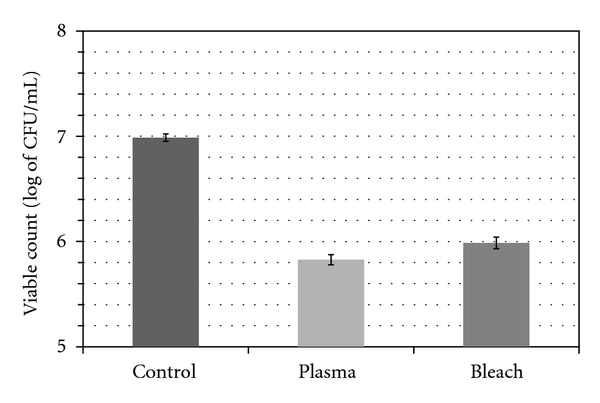
Microbiological analysis: the viable counts (CFU/mL) of *E. faecalis* for all treatment protocols after log transformation (ANOVA, *P* < 0.0001).

The viability of the bacteria on the HA discs was estimated with the CFU analysis. A mean (±SD) CFU mL^−1^ of  9.7 × 10^6^ (±8.1 × 10^5^) (*P* < 0.0001) was obtained for the negative control group (the gas flow-treated group). Treatment of 5.25% NaOCl and the plasma resulted in reduction of the bacterial load to mean (±SD) CFU mL^−1^ of  9.7 × 10^5^ (±1.3 × 10^5^) and 6.7 × 10^5^ (±7.7 × 10^4^) (*P* < 0.0001), corresponding to 90.0% and 93.1% kill, respectively. The results of the CFU mL^−1^ for all groups after log transformation are given in Figure 2, indicating that the plasma treatment is comparable to or slightly more effective than the treatment with 5.25% NaOCl for the same time of exposure.

### 3.2. Morphologic Observations

**Figure 3:**
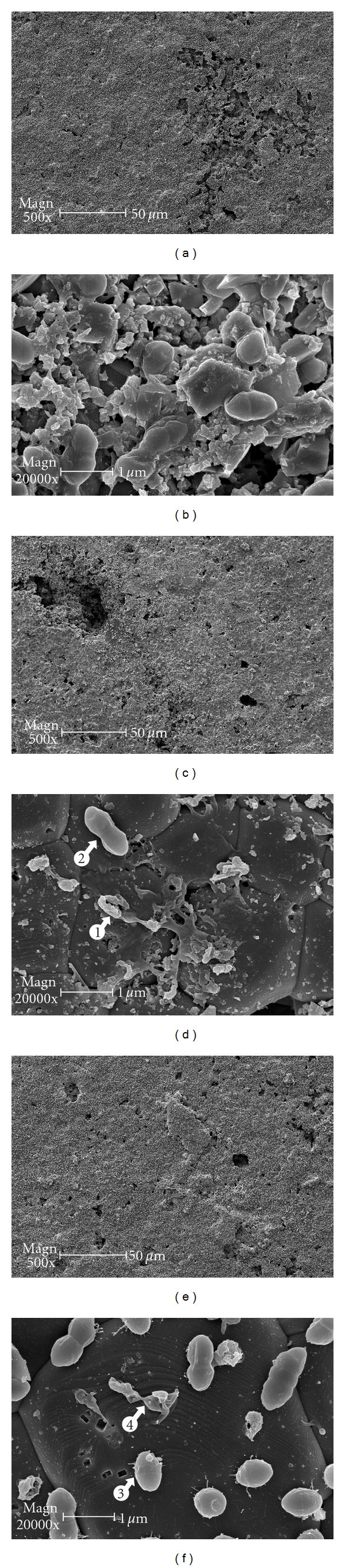
SEM. (a) Uniformly distributed *E. faecalis* biofilms on HA discs after the treatment of gas flow (He/(1%)O_2_, 1 SLPM, 5 min) (the negative control). (b) Biofilms in the negative control group at higher magnification (20,000x): bacteria were adsorbed onto the HA surface and embedded in a self-produced extracellular matrix, as in a typical biofilm. (c) HA surface after direct plasma exposure (He/(1%)O_2_ plasma, 5 SLPM, 5 min). (d) An area of the plasma-treated HA surface at higher magnification (20.000×): debris and fused cell bodies (arrow 1) were predominantly observed. Morphologically intact bacteria (arrow 2) were rarely spotted. (e) HA surface after treatment of 5.25% NaOCl for 5 min (the positive control). (f) Same biofilms of the positive control group at higher magnification (20.000×): most of the remaining bacteria appear morphologically intact (arrow 3). Deformed or fused cell bodies (arrow 4) were discernible but few.

Evenly distributed *E. faecalis* biofilms growing on HA discs of the negative control group (treatment with gas flow only) were observed, as shown in Figure 3(a). A SEM image with higher magnification (Figure 3(b)) revealed biofilm bacteria clinging to the substrate and embedded in the self-produced matrix. It should be remembered that the 1 mm diameter plasma plume was vertically impinging to the center of the HA disc, and the plasma device nozzle was fixed 1 cm above the disc surface (Figure 1(b)). This setup resulted in areas of the discs in the irradiation group never exposed to plasma. Thus, biofilms ultrastructurally similar to those in the control group were observed in the areas, near the edge of the disc or at least 6.5 mm radially away from the disc center, of the plasma treatment group. However, debris and bacteria-free patches were predominantly observed on the HA surface directly under the plasma exposure (Figure 3(c)). Morphologically intact bacteria were rare but detectable (arrow 1 in Figure 3(d)) among the majority of debris and fused cell bodies (arrow 2 in Figure 3(d)). In comparison, the SEM images of the NaOCl-treated *E. faecalis* biofilms showed much more remaining cells with minimum morphology alteration throughout the HA surface (Figure 3(e); arrow 3 in Figure 3(f)), together with few discernible debris (arrow 4 in Figure 3(f)).

## 4. Discussion

The goal of this study was to investigate the antimicrobial effect of a non-thermal plasma against *E. faecalis* biofilms. This *in vitro*, single-species biofilm model was not to closely mimic endodontic biofilms in their natural habitat, which typically consist of numerous species that are arranged in multiple layers [21, 22], but to have a simple and reproducible model system to assess the bactericidal activity of the cold plasma. Sintered HA discs with saliva coating were used as the substrate for biofilm growth. Although a more relevant substrate for the evaluation of endodontic disinfectants might be made from root dentin [23], HA discs offer more controllable properties and easier to standardize. This is important for the first-step assessment of the antimicrobial effects of the cold plasma. In addition, hydroxyapatite is the major mineral content (around 70 wt%) of dentin. A recent microscopic study of endodontic biofilms by Shen et al. [24] showed that HA, with and without coating of type I collagen, provided an excellent substrate for multispecies biofilm growth. In this study, SEM revealed viable layers of *E. faecalis* biofilm colonies evenly formed over the HA discs of the negative control group. The resulted mean CFU mL^−1^, 9.7 × 10^6^, is comparable to previously reported cell densities of *E. faecalis* biofilms [25, 26]. Thus, this *in vitro* test is based on a viable single-species biofilm model.

As the positive control, 5.25% NaOCl irrigation was applied to the biofilm-formed specimens. Numerous research studies have shown that 1–6% sodium hypochlorite solutions are efficient in eliminating *E. faecalis* biofilms compared to many other solutions including 2% chlorhexidinegluconate (CHX), BioPure MTAD, and Tetraclean [26, 27]. It was reported previously that almost complete biofilm removal was achieved after 5-minuate exposure to 5.25% NaOCl [26, 27]. To be comparable, 5-minuate contact time was used in this study. The percentage kill of *E. faecalis *(90%) after 5-min exposure of 5.25% NaOCl was slightly less than the reported results (>99%) [26, 27]. The difference in the reported percentage kills may be due to the different methodology details that it involved. The biofilms used for this study were grown on HA discs for a longer period of time (6 days) compared to those used in the studies by Dunavant et al. (grown on porcelain coupons for one day) [27] or Giardino et al. (grown on membrane filters for 2 days) [26]. The physiological state of the bacteria and the stage of the *E. faecalis*-substrate interactions may influence the susceptibility of *E. faecalis* biofilms to the medicament [11, 28]. After all, treatment of the *E. faecalis* biofilms with 5.25% NaOCl is to provide a reference and to help evaluate the antimicrobial effect of the cold plasma.

The CFU analysis showed that the cold plasma has comparable antimicrobial activity against *E. faecalis* biofilms as 5.25% NaOCl for the same time of contact. In the present proof-of-principle study, while NaOCl had free access to all over the surface distributed by bacteria, the needle-like plasma plume was only applied to a narrow area in the center of the HA disk. Under SEM, most of the surface except near the edge was cleared from the bacterial biofilms throughout the plasma-treated HA samples. Diffusion of the reactive plasma species over a larger surface when the plasma plume came in contact with the dielectric substrate may explain the extended antimicrobial action. Viable bacteria were mostly detected in areas near the edge of the disc or sufficiently far away (e.g., >6.5 mm) from the plasma plume. This demonstrated that the plasma was most effective for surfaces directly under exposure, and the bactericidal effects weakened or diminished for farther distance. Additional to the reduction rate, it must be noted that the plasma demonstrated its potential to remove bacteria from the HA substrate, while the NaOCl left living and dead bacteria at their place. To improve and eventually achieve complete biofilm removal, it is possible to scan the plasma plume over the whole surface area, for example, applying the plasma plume at one spot for 5 min, then move to another 5 mm in-distance spot for another 5 min exposure, and so forth. For endodontic treatment, the accessibility of the plasma plume to complex root canal surfaces must be evaluated and the bactericidal effects against bacterial biofilms under clinic conditions have to be reassessed. Nevertheless, this *in vitro* study is the first step to help optimize the plasma device and evaluate the plasma-mediated bactericidal effect.

## 5. Conclusion

In this proof-of-principle study, the room temperature plasma jet showed comparable antimicrobial effect as 5.25% NaOCl against *E. faecalis* biofilms on HA discs. Better disinfection results may be achieved with scanning of the plasma jet to cover the entire HA surface under treatment. In addition to the demonstrated biofilm-removing effect, the cold plasma may be safer than the conventional medicament irrigation as the enhanced oxidation provided by reactive plasma species is more localized. Nevertheless, more studies are needed to assess the feasibility and effectiveness of the cold plasma-based technology for root canal disinfection. The team is in the process of investigating the bactericidal effect of the plasma jet in the root canal system grown with multispecies endodontic biofilms. The results will be reported in the near future.

##  Conflict of Interests

The authors affirm that there is no potential conflict of interest. There is no financial affiliation or involvement with any commercial organization with direct interest in the subject or materials of the presented work.
